# Insights into *Cedecea neteri* strain M006 through complete genome sequence, a rare bacterium from aquatic environment

**DOI:** 10.1186/s40793-017-0255-1

**Published:** 2017-07-21

**Authors:** Kok-Gan Chan, Wen-Si Tan

**Affiliations:** 1 Division of Genetics and Molecular Biology, Institute of Biological Sciences, Faculty of Science, University of Malaya, 50603 Kuala Lumpur, Malaysia; 2 Vice Chancellor Office, Jiangsu University, Zhenjiang, 212013 People’s Republic of China

**Keywords:** *Cedecea*, Gram-negative, Facultative anaerobic, Genome

## Abstract

*Cedecea neteri* M006 is a rare bacterium typically found as an environmental isolate from the tropical rainforest Sungai Tua waterfall (Gombak, Selangor, Malaysia). It is a Gram-reaction-negative, facultative anaerobic, bacillus. Here, we explore the features of *Cedecea neteri* M006, together with its genome sequence and annotation. The genome comprised 4,965,436 bp with 4447 protein-coding genes and 103 RNA genes.

## Introduction

The *Cedecea* genus is an extremely rare member of the *Enterobacteriaceae* family [1]. The name *Cedecea* was proposed in 1980 for a new genus formerly designated as CDC Enteric Group 15 [1, 2]. *Cedecea* is characterized by positive lipase activity, resistance to colistin and cephalothin, and the inability to hydrolyze gelatin or DNA [3–5]. Discovery was from human sources where its natural environmental habitat remains unknown, *Cedecea* constitutes a rare pathogen of rising importance [6]. To date, only a few species of *Cedecea* have been identified: *C. davisae*, *C. lapagei* and *C. neteri*. All three species exhibit different behaviors in the human body. *C. davisae* has been reported to be associated with scrotal abscess [7] and, most recently, to cause bacteraemia in patients with sigmoid colon cancer [8]. On the other hand, *C. lapagei* has mostly been reported to be involved in pneumonia cases [5, 9]. *C. neteri* is associated with bacteremia in heart disease patients [4] and patients with systemic lupus erythematosus [10].

Strain M006 is a strain of *Cedecea neteri* and is an aquatic isolate from the Sungai Tua Waterfall, a Malaysian tropical rainforest waterfall (N 03 19.91′ E 101 42.15′). In this study, we present an overview of the classification and features of *C. neteri* M006 as well as its genome sequence and annotation. There are a few *C. neteri* aquatic isolates deposited in GenBank and *C. neteri* strain M006 was one of the few isolates discovered from a waterfall which its genome feature has not been reported. Hence, here we firstly reported the genome information of *C. neteri* M006 isolated from a waterfall environment.

## Organisms Information

### Classification and features

**Fig. 1:**
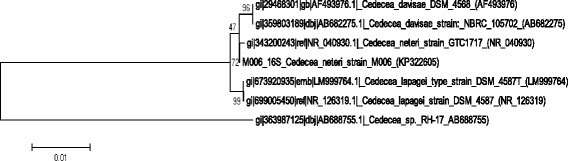
Phylogenetic tree highlighting the position of *Cedecea neteri* M006 relative to the type strains of other species within the genus of *Cedecea*. The strains and their corresponding GenBank accession numbers of 16S rRNA genes are indicated in parentheses. The sequences were aligned and the phylogenetic inferences were obtained using the maximum-likelihood method with MEGA version 5 [12]. The numbers at the nodes are the percentage of bootstrap values obtained by 500 replicates. Bar, 0.01 substitutions per nucleotide positions

Strain M006 was categorized as a member of the genus *Cedecea* by 16S rRNA phylogeny and phenotypic characteristics (Table 1). The EzTaxon database [11] was used as the preliminary 16S rRNA gene sequence-based identification. Strain M006 was most closely related to *C. neteri* GTC 1717T (GenBank accession = AB086230) with a sequence similarity of 99.78%. Subsequent phylogenetic analysis was performed comparing the 16S rRNA gene sequences of strain M006 and related species (Fig. 1). The sequences were aligned and phylogenic trees were built using neighbor-joining (NJ) and maximum-likelihood (ML) methods implemented in MEGA version 5 [12].

**Table 1:** Classification and general features of *Cedecea neteri* M006 according to MIGS recommendations [14]

MIGS ID	Property	Term	Evidence code
	Classification	Domain Bacteria	TAS [22]
		Phylum *Proteobacteria*	TAS [23, 24]
		Class *Gammaproteobacteria*	TAS [25–27]
		Order *unknown*	TAS [23]
		Family *Enterobacteriaceae*	TAS [28–30]
		Genus *Cedecea*	TAS [4]
		Species *Cedecea neteri*	IDA
		Strain: M006	
	Gram stain	negative	TAS [4, 10]
	Cell shape	bacillus	TAS [4, 10]
	Motility	motile	TAS [4]
	Sporulation	Non-spore forming	NAS
	Temperature range	4-28 °C	IDA
	Optimum temperature	28 °C	IDA
	pH range; Optimum	e.g., 5.0-8.0; 7	IDA
	Carbon source	D-sorbitol, Sucrose, D-xylose, malonate	TAS [4]
MIGS-6	Habitat	waterfall	IDA
MIGS-6.3	Salinity	unknown	IDA
MIGS-22	Oxygen requirement	Facultative anaerobic	TAS [4, 10]
MIGS-15	Biotic relationship	Free-living	TAS [4]
MIGS-14	Pathogenicity	Non-pathogen	IDA
MIGS-4	Geographic location	Sungai Tua Waterfall, Malaysia	IDA
MIGS-5	Sample collection	2013	IDA
MIGS-4.1	Latitude	N 03 19.91′	IDA
MIGS-4.2	Longitude	E 101 42.15′	IDA
MIGS-4.4	Altitude	586 m	IDA

Evidence codes – IDA: Inferred from Direct Assay; TAS: Traceable Author Statement (i.e., a direct report exists in the literature); NAS: Non-traceable Author Statement (i.e., not directly observed for the living, isolated sample, but based on a generally accepted property for the species, or anecdotal evidence). These evidence codes are from the Gene Ontology project [31]

**Fig. 2:**
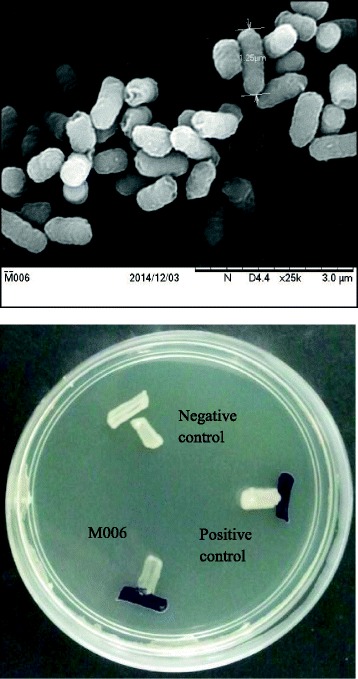
Scanning electron micrograph of *Cedecea neteri* M006. Scale bar 3.0 μm

*C. neteri* M006 cells are Gram-negative, bacillus in shape (0.6-0.7 × 1.3-1.9 μm), are facultatively anaerobic and are motile with 5-9 peritrichous flagella. Colonies formed on nutrient agar are 1.5 mm in diameter and non-pigmented. Scanning electron micrograph pictures of nutrient broth grown cultures showed free-floating cells and clotted cells (Fig. 2). The carbon sources utilized by *C. neteri* are D-sorbitol, sucrose, D-xylose and malonate. *C. neteri* is reported to be unable to utilize dulcitol, adoitol, L-rhamnose, erythritol, glycerol and mucate. The optimal temperature for strain M006 is 28 °C.

**Fig. 3:**
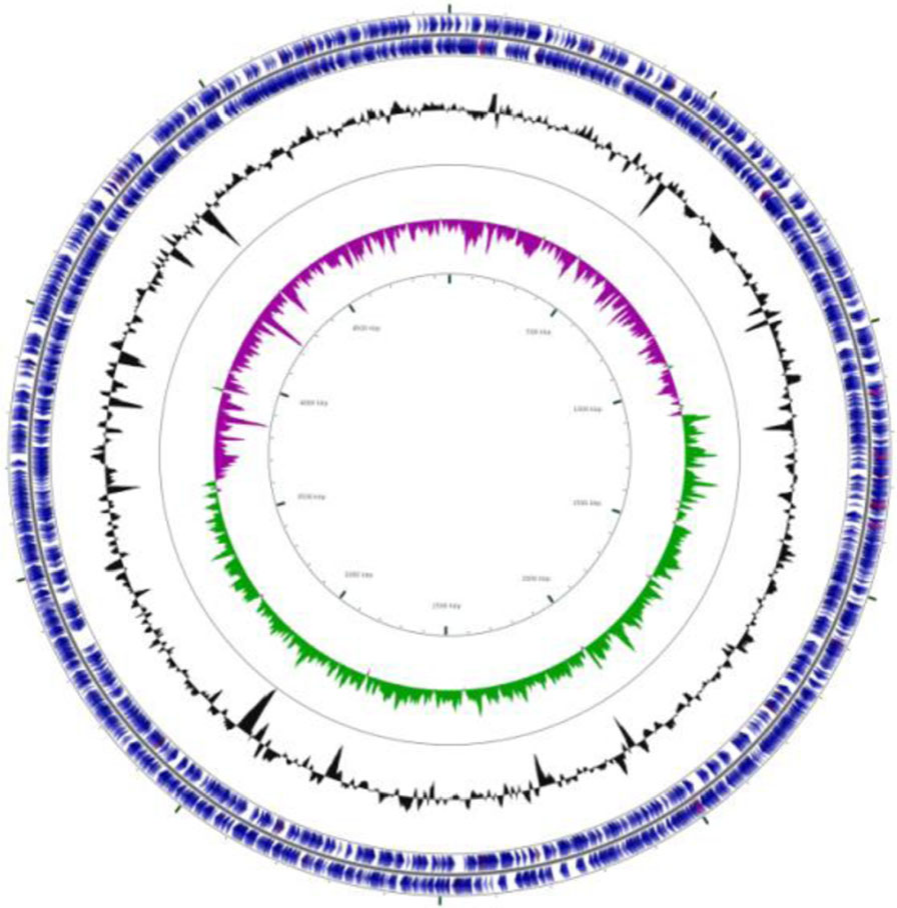
Preliminary screening for AHL. AHL screening of strain M006 with CV026. *E. carotovora* PNP22 and *E. carotovora* GS101 served as negative and positive controls respectively

*C. neteri* M006 cells are Gram-negative, bacillus in shape, survive facultative anaerobically and are motile. The colonies formed on nutrient agar are 1.5 mm in diameter and are non-pigmented. The colony is whitish in color and the appearance is round with a smooth edge. Signaling molecules, known as *N*-acylhomoserine lactone, are produced for communication purposes in order to regulate physiological properties. The preliminary screening of strain M006 using the bacterial biosensor *Chromobacterium violaceum* (CV026) showed the purple pigmentation indicative the presence of signaling molecules (Fig. 3).

## Genome sequencing information

### Genome project history

Strain M006 was selected for the sequencing based on its phylogenetic position and the similarity of its 16S rRNA to other members of the genus *Cedecea*, The genome project was deposited in the Genomes On-Line Database [13] and the genome sequence was deposited in GenBank (CP009458.1). A summary of the project and the Minimum Information about a Genome Sequence (MIGS) [14] are shown in Table 2.

**Table 2:** Genome sequencing project information

MIGS ID	Property	Term
MIGS 31	Finishing quality	Complete
MIGS-28	Libraries used	PacBio
MIGS 29	Sequencing platforms	PacBio
MIGS 31.2	Fold coverage	74.34×
MIGS 30	Assemblers	HGAP V 2.1.1
MIGS 32	Gene calling method	IMG-ER
	Locus Tag	LH23
	Genbank ID	CP009458
	Genbank Date of Release	2014/10/22
	GOLD ID	Gp0109502
	BIOPROJECT	PRJNA260769
MIGS 13	Source List Identifier	M006
	Project relevance	Environmental

### Growth conditions and genomic DNA preparation

*Cedecea neteri* M006 was cultured aerobically on Luria-Bertani (LB) agar medium at 28 °C overnight (16-18 h). Genomic DNA was extracted using the MasterPure™ DNA Purification Kit (Epicentre Inc., Madison, WI, USA). The extracted genomic DNA was examined via a NanoDrop spectrophotometer (Thermo Scientific, Waltham, MA, USA) and a Qubit 2.0 fluorometer (Life Technologies, Carlsbad, CA, USA) for its quality.

### Genome sequencing and assembly

The genome of strain M006 was sequenced at the microbiome lab, High Impact Research, University Malaya, using a Pacific Biosciences single-molecule real-time (PacBio SMRT) sequencer. The sequencing was carried out using P5 chemistry on two SMRT cells with a 20-kb prepared SMRTbell library [15]. De novo assembly of 41,094 reads using the hierarchical genome assembly process in the SMRT version 2.1.1 portal resulted with one contig of 3.96 Mb in size. The sequencing average coverage is 74.34 × and this genome has a GC content of 54.41%.

### Genome annotation

After genome assembly, it was analyzed using Rapid Annotation using Subsystem Technology server databases (version 2.0) [16], which identified 4423 predicted coding sequences with a total of 103 RNA genes. The predicted open reading frames were annotated by searching clusters of orthologous groups [17] using the Integrated Microbial Genomes Expert Review [18]. The different groups of RNAs (rRNA and tRNA) were identified by using RNAmmer 1.2 [19] and tRNAscan-SE 1.23 [20] respectively. The additional gene prediction analysis and functional annotation were performed within IMG-ER platform.

## Genome properties

**Fig. 4:**
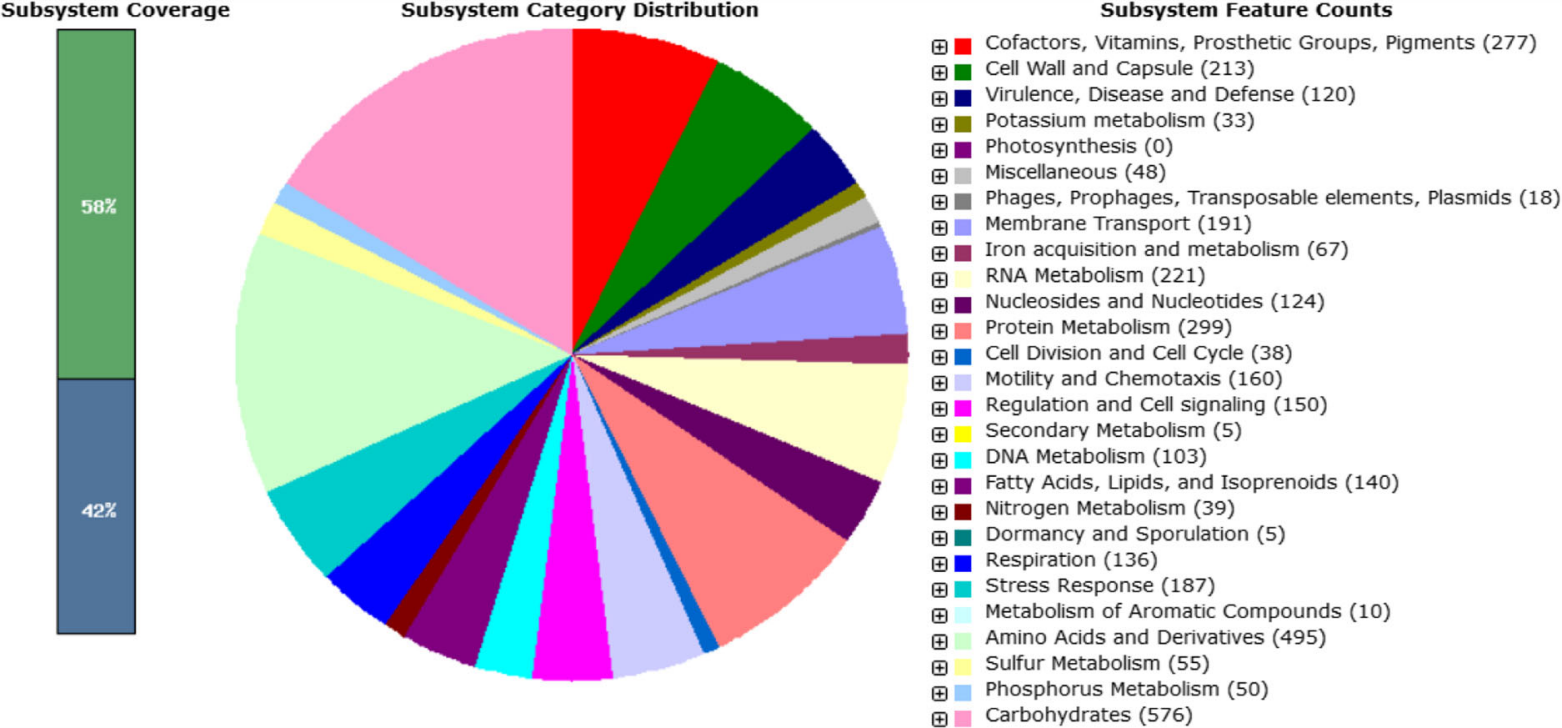
Graphical circular map of the genome. Starting from the outermost circle and moving inwards, each ring of the circle contains information on a genome: tRNA/rRNA, genes on the reverse and forward strands, GC skew and GC ratio

The genome comprised a circular chromosome with a length of 4,965,436 bp and 54.41% G + C content (Fig. 4 and Table 3). It is composed of one contig and of the 4550 predicted genes, 4447 were protein-coding genes. The properties of and the statistics for the genome are summarized in Table 3. The distribution of genes into COG functional categories is presented in Table 4.

**Table 3:** Genome statistics

Attribute	Value	% of total
Genome size (bp)	4,965,436	100
DNA coding (bp)	4,350,834	87.62
DNA G + C (bp)	2,701,616	54.41
DNA scaffolds	1	100
Total genes	4550	100
Protein coding genes	4447	97.74
RNA genes	103	2.26
rRNA genes	22	0.48
tRNA	80	1.76
Pseudo genes	24	0.53
Genes in paralog clusters	3462	76.09
Genes with function prediction	4091	89.91
Genes assignmed to COGs	3611	79.36
Genes with Pfam peptides	4095	90.00
Genes with signal peptides	466	10.24
Genes with transmembrane helices	1079	23.71
CRISPR repeats	0	0.00

**Table 4:** Number of genes associated with general COG functional categories

Code	Value	% age^a^	Description
J	189	4.70	Translation, ribosomal structure and biogenesis
A	1	0.02	RNA processing and modification
K	395	9.82	Transcription
L	133	3.31	Replication, recombination and repair
B	0	0.00	Chromatin structure and dynamics
D	32	0.80	Cell cycle control, Cell division, chromosome partitioning
V	47	1.17	Defense mechanisms
T	181	4.50	Signal transduction mechanisms
M	224	5.57	Cell wall/membrane biogenesis
N	117	2.91	Cell motility
U	105	2.61	Intracellular trafficking and secretion
O	145	3.60	Posttranslational modification, protein turnover, chaperones
C	231	5.74	Energy production and conversion
G	362	9.00	Carbohydrate transport and metabolism
E	412	10.24	Amino acid transport and metabolism
F	96	2.39	Nucleotide transport and metabolism
H	158	3.93	Coenzyme transport and metabolism
I	109	2.71	Lipid transport and metabolism
P	266	6.61	Inorganic ion transport and metabolism
Q	75	1.86	Secondary metabolites biosynthesis, transport and catabolism
R	409	10.16	General function prediction only
S	337	8.37	Function unknown
-	939	20.64	Not in COGs

^a^The total is based on the total number of protein coding genes in the annotated genome

## Insights from the genome sequence

**Fig. 5:**
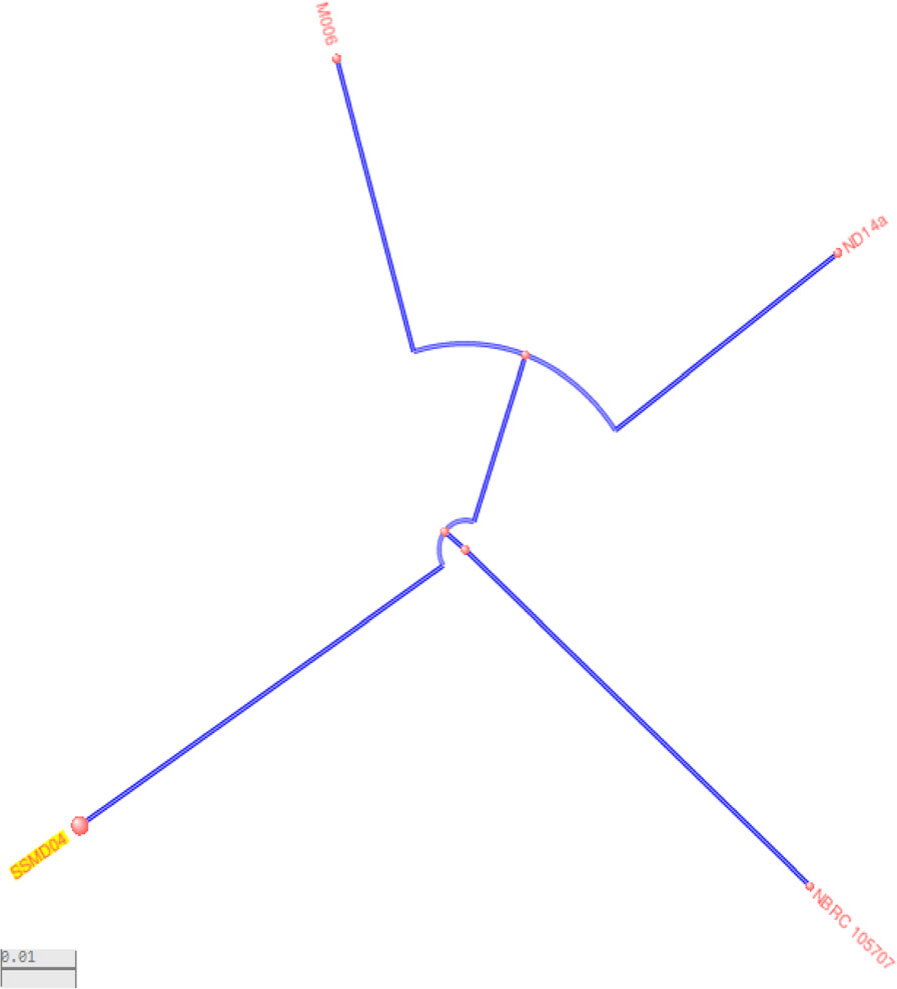
RAST annotation of *C. neteri* strain M006. This annotation pipeline allows a view of the subsystem category distribution of *C. neteri* strain M006. Genes responsible for QS activity in this strain can be found in regulation and cell signaling subsystem (*red arrow*)

RAST annotation allowed the insight of subsystem category distribution of *C. neteri* strain M006. This category enabled the understanding of various functional roles such as protein classes, amino acid biosynthesis and metabolic pathways. There are 552 subsystems. The most abundant subsystem feature belonged to carbohydrate metabolism (*n* = 576; out of a total of 3760 subsystem feature counts), followed by amino acid and derivatives (*n* = 495) and protein metabolism (*n* = 299) (Fig. 5). One of the subsystem features grouped as regulation and cell signaling was focused to allow functional genes related to quorum sensing (QS) activity to be searched. The *in-silico* study identified the novel LuxIR homologue of *C. neteri*, which was later designated as CneIR. The complete open reading frame of *C. neteri* strain M006 *cneI* and *cneR* homologues were found and are 462 bp and 723 bp, respectively. The complete genome sequencing allows deeper understanding of the genetic makeup that may help in identifying the linkage of pathogenicity and virulence factors with its QS properties [15].

**Fig. 6:**
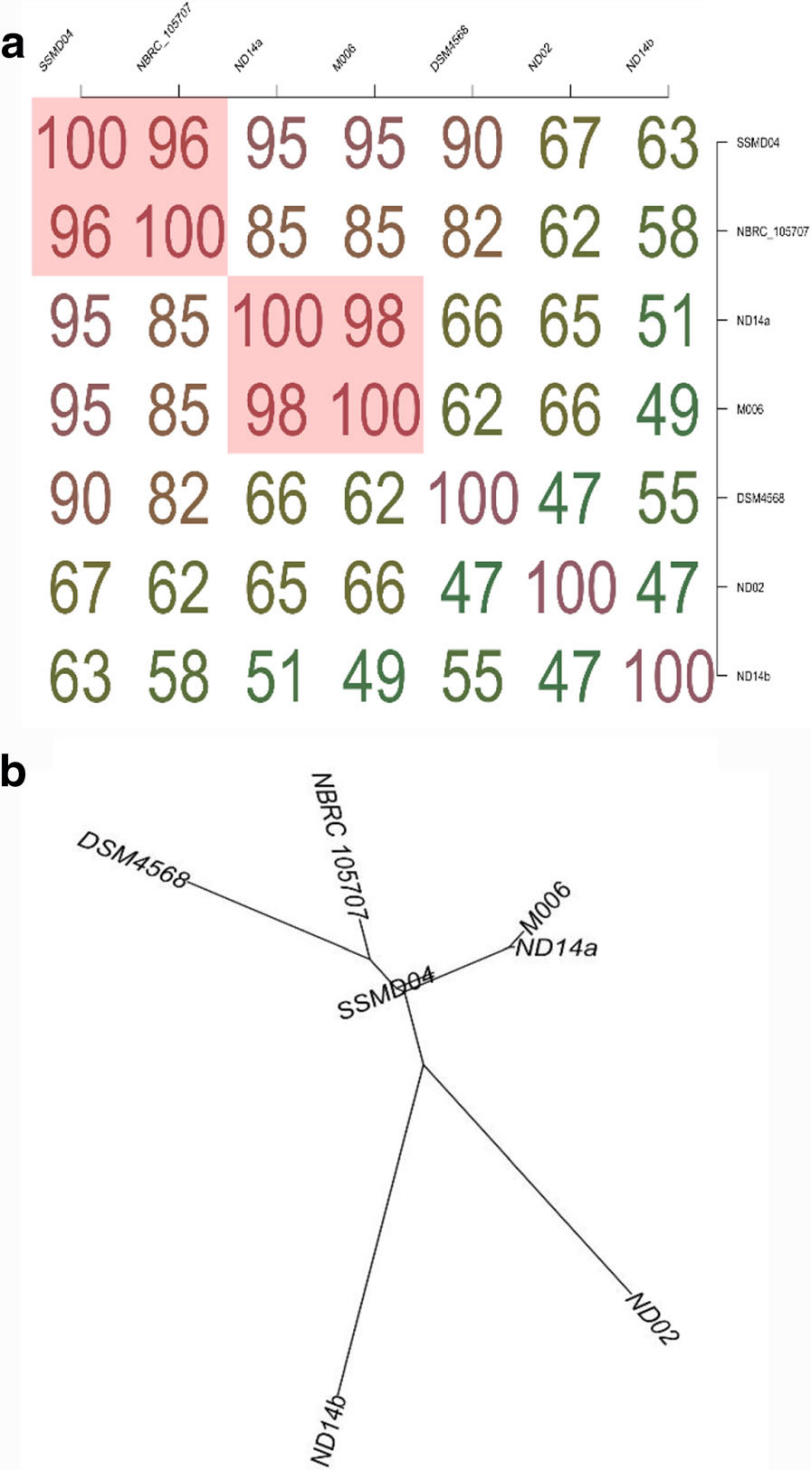
AAI calculation for 6 *C. neteri* strains and 1 *C. davisae* strain. Analyses of conserved genes in the core genome computed based on AAI calculator provided (**a**) an AAI matrix; and (**b**) AAI-based phylogenetic distance tree, clustered according to distance pattern. The AAI-distance tree was clustered based on BIONJ method

Currently, the availability of genomes of this genus is low. Only 5 complete genomes of *C. neteri* strains including strain M006 and a draft genome of type strain NBRC 105707 are deposited in NCBI. A matrix and dendrogram were generated based on AAI calculation that provide estimation of the average amino acid identity using best hits (one-way AAI) and reciprocal best hits (two-way AAI) between several genomic datasets of proteins [21], *C. davisae* type strain DSM 4568 was included in the analyses. From the analyses, we can see closer protein clustering between strain M004 and strain ND14a (Fig. 6). Some of the basic comparisons of the genomes are listed in Table 5.

**Table 5:** Comparison of several strains of *C. neteri*

Organism/Name	Strain	Size (Mb)	GC%	Gene	Protein
*C. neteri*	M006	4.97	54.40	4703	4531
	ND02	4.31	53.90	4053	3884
	ND14b	5.05	56.90	4491	4295
	ND14a	4.66	54.80	4426	4215
	SSMD04	4.88	55.10	4622	4416
	NBRC 105707	5.20	54.10	4944	4739

## Conclusion

This study provides phenotypic and genomic insights into *Cedecea neteri* strain M006. It reports the isolation of *C. neteri* from an aquatic environment for the first time. This study also revealed of the QS ability of *C. neteri*.

## Abbreviations

AHL: *N*-acylhomoserine lactone
HGAP: hierarchical genome assembly process
LB: Luria-Bertani
ML: Maximum-likelihood
NJ: Neighbor-joining
QS: Quorum sensing
RAST: Rapid Annotation using System Technology
